# Relationship between Risk Behavior for Eating Disorders and Dental Caries and Dental Erosion

**DOI:** 10.1155/2017/1656417

**Published:** 2017-12-20

**Authors:** Lorenna Mendes Temóteo Brandt, Liege Helena Freitas Fernandes, Amanda Silva Aragão, Yêska Paola Costa Aguiar, Sheyla Márcia Auad, Ricardo Dias de Castro, Sérgio D'Ávila Lins Bezerra Cavalcanti, Alessandro Leite Cavalcanti

**Affiliations:** ^1^Department of Dentistry, State University of Paraíba, Campina Grande, PB, Brazil; ^2^Department of Dentistry, Faculty of Dentistry, Federal University of Minas Gerais, Belo Horizonte, MG, Brazil; ^3^Department of Dentistry, Federal University of Paraíba, João Pessoa, PB, Brazil

## Abstract

The aim of this study was to evaluate whether there is an association between risk behavior for eating disorders (EDs) and dental erosion and caries. A controlled cross-sectional study was conducted in Brazil, involving 850 randomly selected female adolescents. After evaluating risk behavior for eating disorders through the Bulimic Investigatory Test of Edinburgh, 12 adolescents were identified with severe risk behavior for EDs and matched to 48 adolescents without such risk. Dental examinations, anthropometric measurements, and eating habits and oral hygiene were performed. Adolescents with high severity eating disorder condition were not more likely to show dental caries (*p* = 0.329; OR = 2.2, 95% CI: 0.35–13.72) or dental erosion (*p* = 0.590; OR = 2.33; 95% CI: 0.56–9.70). Adolescents with high body mass index (BMI) were five times more likely to have high severity eating disorder condition (*p* = 0.031; OR = 5.1; 95% CI: 1.61–23.07). Therefore, high severity risk behavior for EDs was not significantly associated with dental caries and dental erosion. However, high BMI was a risk factor for developing eating disorders and should be an alert for individuals with this condition.

## 1. Introduction

Nowadays, especially among women, there is a constant concern with body weight, which is a central issue present in different social segments. This behavior has obtained special support from the media that constantly shows individuals with images of ideal thinness based on processes such as the adherence of women to constant and stubborn search for a body shape considered beautiful [1].

The etiology explaining the genesis and maintenance of eating disorders is multifactorial, involving biological, social, and psychological factors, and the key factor is the distorted self-perception and dissatisfaction with physical appearance [2, 3].

Anorexia nervosa (AN) is the third most common chronic disease among adolescents, and bulimia nervosa (BN) affects over 1% of female adolescents [4]. These two types of eating disorders affect mainly adolescent and young adult women [1, 3]. These eating disorders demonstrate harmful effects on oral health [5–7]. The main manifestations are dental erosion, which is the most often condition associated with eating disorders [5, 8, 9], especially when there is purge habits [5, 6] and caries lesions.

The early diagnosis of eating disorders is important not only due to psychological and somatic complications, but also due to damage to oral health, since they are the only complications that cannot be reversed [8, 10]. The dentist is a professional with potential to suspect about this probable diagnosis based on oral signals and symptoms and can perform the correct multiprofessional referral [5].

Therefore, a theoretical background on eating disorders and their effects on oral health may help the dentist's diagnosis and intervention and correct referral to other health professionals and can thus contribute to improving the quality of life of patients [5, 10].

Despite the increasing prevalence of these disorders, literature has only few studies evaluating the association between eating disorder risk behavior and dental erosion and caries [5, 9]. However, due to the relevance of the theme, there are already two systematic reviews [6, 7], but most studies were conducted with individuals who were already diagnosed with the disease [1, 5].

In this context, this study has investigated whether there is an association between risk behavior for eating disorders and dental erosion and caries among female adolescents, aiming to foster foundation for subclinical early diagnosis among young people.

## 2. Materials and Methods

### 2.1. Ethical Aspects

This research was approved by the Ethics Committee of the State University of Paraíba and the participants were informed about the purpose and methodology of the study and signed a consent form.

### 2.2. Design

This cross-sectional study was conducted in public and private high schools of Campina Grande, Paraiba, Brazil, northeastern Brazil, a city with about 385,213 inhabitants, divided into six health districts, with Human Development Index (HDI) of 0.72 [11].

### 2.3. Sampling

The population of this study totaled 14.351 adolescents and the sample size was calculated using a margin of error of 1%, confidence level of 95%, and prevalence of high severe risk behavior for eating disorders of 1.7% [8] and applying a correction factor of 1.2 because it is a multistage study. The minimum sample size needed to meet the requirements was estimated at 780 individuals. However, an additional 10% were invited to participate in the study in order to compensate losses and refusals, resulting in 858 individuals. This study included 850 adolescents.

### 2.4. Exclusion Criteria

Adolescents who were already diagnosed with eating disorders and gastroesophageal reflux or who used orthodontic braces were excluded from the study.

### 2.5. Calibration and Training Process

Dental examinations were performed by two trained and calibrated examiners: one for the diagnosis of dental caries, according to criteria established by WHO [12], and another for the diagnosis of dental erosion, according to criteria proposed by O'Sullivan et al. [13, 14]. The examiner was calibrated and trained with a gold standard examiner, which is an experienced epidemiologist. There was a theoretical and a practical stage. The examiner and the gold standard conducted clinical trials in 20 adolescent volunteers from a public school to evaluate the interexaminer agreement. Adolescents were examined again after a 15-day interval for the calculation of the intraexaminer agreement. The Kappa Cohen coefficient was calculated for tooth-by-tooth basis. The interexaminer agreement obtained value of 0.97 and intraexaminer agreement obtained value of 0.98.

Calibration for the diagnosis of erosion also consisted of two stages. In the first, the gold standard examiner discussed the codes and criteria of dental erosion, based on the O'Sullivan index [13], with the examiner to be calibrated (YPCA). In the second stage,* in lux* calibration was conducted by projecting 75 images and obtained interexaminer agreement of 0.82 and intraexaminer agreement of 0.74 (after a 15-day interval).

The pilot study was conducted with 59 adolescents to test the methods and the data collection process, demonstrating that there was no need for modifications. Individuals who participated in this stage were not included in the main study.

### 2.6. Data Collection

Data collection was performed by two examiners and four scorers. Initially, the Bulimic Investigatory Test of Edinburgh (BITE) was applied [14], which was validated to be applied in adolescents of the Brazilian population [15] and a sociodemographic questionnaire.

The BITE presents two scales as final results, one of symptoms and another of severity. The symptom scale has three possible outcomes [14] (see Table 1).

The severity scale contains the six items measuring the severity of the disorder as defined by its frequency, and it presents three possible results of the eating disorders: mild severity (less than 5 points); moderate severity (from 5 to 9 points); high severity (from 10 points). It is recommended that participants complete the questionnaire considering their behavior in the past three months [14].

After analysis of BITE, adolescents with eating disorder condition in the BITE symptom scale (score ≥ 20) and high severity on the severity scale (scores ≥ 10) were matched for age and type of school with adolescents without such risk (1 : 4) for the performance of dental examinations. The cutoff point used was based on a previous study [6]. Overall, 12 adolescents had high scores on both scales. Thus, 12 adolescents at high severity eating disorder condition and 48 with normal eating behaviors were submitted to clinical examination in order to verify the occurrence of dental erosion and caries (*n* = 60). Prior to clinical examination, adolescents were submitted to weight and height measurement to calculate body mass index (BMI).

Dental examinations were conducted in private rooms provided by the schools. Adolescents were positioned face to face with the examiner. At this stage, all appropriate personal protective equipment (PPE) was used. Examinations were performed under artificial lighting (headlamp Petzl Zoom, Petzl America, Clearfield, UT, USA) with mouth mirrors (PRISMA®, São Paulo, SP, Brazil) packaged and sterilized, and sterile gauze pads (used to clean and dry the teeth), in line with infection control regulations [12]. The blinding process was used by examiners.

### 2.7. Statistical Analysis

Data were analyzed using the Statistical Package for Social Sciences (SPSS for Windows, version 18.0, SPSS Inc., Chicago, IL USA). Descriptive statistical analysis (frequency and distribution) was performed. Bivariate analyses were performed to test the association between high severity eating disorder condition and dental erosion and caries, high severity eating disorder condition, and sociodemographic variables and physical aspects, using the exact versions of the Pearson chi-square and Fisher's exact tests. A conditional logistic regression model (backward) was used to determine the association of independent variables with high severity eating disorder condition. Independent variables were included in the conditional logistic model based on their statistical significance in the bivariate analysis (*p* < 0.20). The statistical significance level was set at 5%, with 95% confidence interval.

## 3. Results

The sample consisted of 850 adolescents aged 15–18 years (34.1% aged 15 years, 33.1% aged 16 years, 23.2% aged 17 years, and 9.6% over 18 years). Most participants (76.2%) were public school students, who had not used dental services in the last six months (56.0%) and monthly family income above one minimum wage (56.1%) (Table 2). The BITE symptom scale identified 41 adolescents (4.8%) with score equal to or above 20, indicating high likelihood of presenting bulimia nervosa (Table 2), and 12 (1.4%) reached the highest cutoff points both in the symptom scale and in the severity scale, indicating not only the possibility of meeting the criteria of bulimia, but also the presence of a high severity eating disorder condition.

The prevalence of dental erosion and caries in the sample was 10.0% and 60.0%, respectively. Dental examinations showed that 75.0% of adolescents with high severity eating disorder condition had dental caries, and 56.2% belonging to the no risk group had the same condition (*p* = 0.329). With regard to dental erosion, the proportions for these groups were 16.7% and 8.3%, respectively (*p* = 0.590) (Table 3).

When testing the association between high severity eating disorder condition and sociodemographic variables and physical aspects, statistically significant difference was observed regarding the use of dental services in the last six months (*p* < 0.05) and BMI (*p* < 0,05) (Table 4). Conditional logistic regression showed that adolescents with high BMI (overweight or obese) were more likely to have high severity eating disorder condition when controlled by independent variable use of dental services (*p* = 0.031; OR = 5.1; 95% CI = 1.61–23.07) (Table 5).

## 4. Discussion

This study included only female adolescents, similar to other studies [5, 8, 9]. Generally, studies on eating disorders recruit patients already diagnosed with this condition and referred for medical and/or psychological treatment [10]. Importantly, participants of this population-based study came from a student population and have been identified and selected through a validated instrument [14] widely recognized in the literature used to detect binge eating behavior, assessing cognitive and behavioral aspects related to bulimia nervosa. In the research involving hospitalized patients, it is more likely to include individuals with more severe eating disorders and more serious dental implications [10], which should be taken into account when comparing and discussing the results of this investigation.

Another relevant aspect is that this study adopted the blinding of examiners in relation to the condition of each adolescent regarding the presence of risk behavior for bulimia nervosa. Few studies in the literature that have investigated eating disorders and oral health conditions [10, 16] have used the blinding process during data collection phase. This process is of utmost importance and aims to reduce possible measurement bias.

The identification of adolescents at risk for eating disorders in this study was associated not only to cutoff points of the BITE symptom scale (≥20), but also to the severity scale (≥10). As a result, this sample can be characterized as a group with high-risk behavior suffering from BN [6].

Thus, according to the BITE symptom scale, this study found 41 adolescents (4.8%) with a score equal to or greater than 20, indicating a high chance to meet the criteria for BN. Other Brazilian studies using the same survey instrument have found prevalence of 1.1% [17], 1.7% [5], and 6.0% [8]. Importantly, this different prevalence related to two of these studies [5, 17] may be linked to the fact that they have included students of different age groups, 7–19 years [17] and 12–16 years [5] of both sexes, making comparisons limited. It is also known that eating disorders affect mainly female adolescents and there has been an increase in the high-risk group aged 15–19 years [18]. This fact explains the higher prevalence of risk behavior for eating disorders found both in this and in a previous study [8], compared with studies involving both sexes and less restricted age groups.

Among the 41 participants, 12 have achieved the highest cutoff points from both subscales, indicating not only the high chance to meet the bulimia criteria but also a condition with a high severity. Among these 12 participants, 66.6% were from public schools and 33.3% from private schools. However, students from private schools had proportionately more cases of risk behavior for eating disorders than those from public schools, corroborating previous findings [8]. In Brazil, students enrolled in private school tend to have higher income compared to students enrolled in public schools and may suggest that there is a correlation between eating disorders and income, as demonstrated both in this and in another study [8]. Some authors reported that the prevalence of these diseases is lower in less developed areas [17].

In this study, there was no association between dental erosion and high severity eating disorder condition, although 66.6% of the risk adolescents were active purging, unlike results found by other Brazilian researchers [5, 8] and other studies conducted in other countries with different populations and methods, which indicated a possible causal relationship between eating disorders and dental erosion [10, 19]. The temporality of disorder presence is correlated with dental erosion [19], but it takes a continuous contact of acids with dental tissue for erosion to occur. Some authors have suggested that the biofilm can be a possible protective factor against acid attacks and the development of dental erosion [20]. The lack of association between dental erosion and high severity eating disorder condition in this study may have occurred because the teenagers have been framed at grave risk not long ago.

The lack of association between dental erosion and adolescents with high severity eating disorder condition may also be related to the low prevalence of dental erosion in the group of high severity eating disorder condition and to the presence of erosion among the no risk group. This fact can be explained by the increased incidence of this condition in the population due to changes in lifestyle and increase in the total value and frequency of consumption of products containing acids [21–23].

This study revealed no association between high severity eating disorder condition and dental caries, confirming previous findings [5, 8]. However, a previous study has shown that individuals suffering from bulimia had higher retention of dental plaque and reduced salivary flow, which could be associated with the occurrence of dental caries [24]. A possible explanation for this lack of association in studies conducted in Brazil may be the overall high prevalence of dental caries in the Brazilian population [25, 26]. Another possible explanation is that in this study we have used a research instrument (BITE) as proxies variable to bulimia, unlike the study by Paszyńska et al. [24] that found the grievance in individuals already diagnosed with bulimia.

One of the limitations of the study was the use of the WHO criteria for the diagnosis of dental disease. This method does not allow the diagnosis of initial enamel lesions, allowing only the registration of lesions cavitated in dentin [27]. However, the CPO-D index is established by WHO and used by several studies.

In this study, it was observed that most adolescents with high severity eating disorder condition (83.3%) did not undergo routine dental examinations, unlike the no risk group in which more than half of the adolescents (58.3%) underwent dental examinations (*p* < 0.05). This fact can be explained because patients with eating disorders usually avoid contact with health professionals, hiding the true source of the problem, by either guilt, shame, or even denial of their condition [18].

There were a higher proportion of individuals with BMI higher than normal among individuals with high severity eating disorder condition compared to no risk group, which suggests that individuals with high severe risk behavior for eating disorders have binge eating behavior followed by inappropriate compensatory acts as a way to compensate their overweight. In a previous study [28], the criteria used for university students to feel satisfied with their appearance were associated with weight (*p* < 0.05). Body mass index is regarded as an important predictor of body satisfaction and related behaviors [29].

Logistic regression analysis revealed that overweight or obese adolescents are more likely to have serious risk behavior for eating disorders, corroborating previous findings [30, 31]. Thus, it was observed that higher BMI leads to greater dissatisfaction with appearance and increased prevalence of eating disorders [31].

Female adolescents who are only suspected or just identified with severe risk behaviors for EDs probably cannot represent a high-risk group with dental side effects in hard dental tissues. This is a positive issue for dentists that time needed for development of dental complications is extended to long period of time. Therefore, medical/dental interventions to stop risk behaviors have longer time to prevent against oral complications.

## 5. Conclusion

It was observed that there was no association of dental caries and dental erosion in adolescents with high severity eating disorder condition. Adolescents with high BMI should be followed, since they are individuals with potential for developing eating disorders. In addition, longitudinal studies should be carried out in order to clarify the temporality of the presence of eating disorders and dental diseases.

## Figures and Tables

**Table 1:** BITE's symptoms scale.

Outcome	Score
No risk for the development of eating disorders	Less than 10 (score < 10)

Risk situation for the development of eating disorders, which suggests an unusual eating pattern without the presence of all criteria for eating disorder	Score ≥ 10 and less than 20

Eating disorder condition indicating presence of compulsive eating behavior and high possibility of presence of bulimia	Scores of 20 to a maximum of 30

**Table 2:** Sample characterization.

Characteristics	Variable	*n* (%)
Sociodemographic	*Age *[850]	
	15 years	290 (34.1%)
	16 years	281 (33.1%)
	17 years	197 (23.2%)
	18 years	82 (9.6%)
	*Type of school *[850]	
	Public	648 (76.2%)
	Private	202 (23.8%)
	*Dental visit in the last 6 months *[850]	
	Yes	374 (44.0%)
	No	476 (56.0%)
	*Monthly family income *[294]	
	Less than or equal to 1 minimum wage^*∗*^	129 (43.9%)
	Greater than 1 minimum wage	165 (56.1%)

Behavioral and cognitive	*Symptoms of eating disorder (ED) *[850]	
	No risk (normal eating standard)	493 (58.0%)
	Risk situation (nonusual eating habits)	316 (37.2%)
	Situation of eating disorder (presence of binge eating behavior with great chances of having bulimia nervosa)^*∗*^	41 (4.8%)
	*Risk behavior for ED based on symptom severity scales *[41]	
	Not significant (score ≤ 4)	11 (26.9%)
	Clinically significant (score: 5–9)	18 (43.9%)
	High severity (score ≥ 10)	12 (29.2%)

^*∗*^Minimum wage value at the time of the survey: U$ 250.15 in the year 2015.

**Table 3:** Statistical significance and prevalence of dental erosion and caries (*n* = 60).

Groups	Variable			*p* value
	*Dental caries*			
	Present	Absent	Total	
*High severity EDC*	9 (75.0%)	3 (25.0%)	12 (100.0%)	0.329^*∗*^
*No risk*	27 (56.2%)	21 (43.8%)	48 (100.0%)	
	*Dental erosion*			
	Present	Absent	Total	
*High severity EDC*	2 (16.7%)	10 (83.3%)	12 (100.0%)	0.590^*∗*^
*No risk*	4 (8.3%)	44 (91.7%)	48 (100.0%)	

EDC = eating disorder condition; ^*∗*^Fisher's exact test.

**Table 4:** Relationship between high severity eating disorder condition, sociodemographic variables, and physical aspects.

Variables	High severity eating disorder condition		*p* value
	Present	Absent	
*Monthly family income *[*30*]			0.372^*∗*^
Less than or equal to 1 MW^*∗∗*^	1 (16.7%)	10 (41.7%)	
Greater than 1 MW	5 (83.3%)	14 (58.3%)	
Total	6 (100.0%)	24 (100.0%)	

*Use of dental services in the last six months *[*60*]			**0.010**
Yes	2 (16.7%)	28 (58.3%)	
No	10 (83.3%)	20 (41.7%)	
Total	12 (100.0%)	48 (100.0%)	

*Body mass index (BMI) *[*60*]			**0.009** ^**∗**^
Under normal or normal	6 (50.0%)	42 (87.5%)	
Above normal	6 (50.0%)	6 (12.5%)	
Total	12 (100.0%)	48 (100.0%)	

^*∗*^Fisher's exact test. ^*∗∗*^Minimum wage value at the time of the survey U$ 250.15. Statistical significance in bold (*p* < 0.05).

**Table 5:** Conditional logistic regression (stepwise backward entry method), eating disorder condition with high severity with independent variables.

Variable	OR (CI)	*p* value
BMI	5.1 (1.61–23.07)	0.031^*∗*^
Use of dental services in the last six months	5.3 (1.0–28.845)	0.050

Variables incorporated in the model (^*∗*^*p* < 0.20).
